# DNA damage response and DNA repair – dog as a model?

**DOI:** 10.1186/1471-2407-14-203

**Published:** 2014-03-19

**Authors:** Nicole Grosse, Barbara van Loon, Carla Rohrer Bley

**Affiliations:** 1 Division of Radiation Oncology, Vetsuisse Faculty, University of Zurich, Winterthurerstrasse 260, 8057 Zurich, Switzerland; 2 Institute for Veterinary Biochemistry and Molecular Biology, Vetsuisse Faculty, University of Zurich, Winterthurerstrasse 190, 8057 Zurich, Switzerland

**Keywords:** Canine and human tumors, DNA damage response, DNA repair

## Abstract

**Background:**

Companion animals like dogs frequently develop tumors with age and similarly to human malignancies, display interpatient tumoral heterogeneity. Tumors are frequently characterized with regard to their mutation spectra, changes in gene expression or protein levels. Among others, these changes affect proteins involved in the DNA damage response (DDR), which served as a basis for the development of numerous clinically relevant cancer therapies. Even though the effects of different DNA damaging agents, as well as DDR kinetics, have been well characterized in mammalian cells *in vitro*, very little is so far known about the kinetics of DDR in tumor and normal tissues *in vivo*.

**Discussion:**

Due to (i) the similarities between human and canine genomes, (ii) the course of spontaneous tumor development, as well as (iii) common exposure to environmental agents, canine tumors are potentially an excellent model to study DDR *in vivo*. This is further supported by the fact that dogs show approximately the same rate of tumor development with age as humans. Though similarities between human and dog osteosarcoma, as well as mammary tumors have been well established, only few studies using canine tumor samples addressed the importance of affected DDR pathways in tumor progression, thus leaving many questions unanswered.

**Summary:**

Studies in humans showed that misregulated DDR pathways play an important role during tumor development, as well as in treatment response. Since dogs are proposed to be a good tumor model in many aspects of cancer research, we herein critically investigate the current knowledge of canine DDR and discuss (i) its future potential for studies on the *in vivo* level, as well as (ii) its possible translation to veterinary and human medicine.

## Background

Mutations in important driver genes, arising from various defects in the DNA damage response (DDR) pathways, can influence the tumor response to treatment. Hence, affected DDR pathways were a basis for the development of numerous clinically relevant cancer therapies. The effects of different DNA damaging agents, as well as DDR kinetics have been well characterized in mammalian cells *in vitro*. However, very little is known about the amount of actual DNA damage and the kinetics of DDR in tumors, as well as normal tissues *in vivo* under antineoplastic treatment.

Only few studies utilized individual patient material, and initial DNA damage detection in patient tumor cells was rarely performed. Use of lymphocytes irradiated outside of the patient (*ex corpora*) [1,2] revealed individual patient heterogeneity and displayed more background DNA damage in cancer patients vs. healthy individuals [3,4]. Lymphocytes from human head and neck squamous cell carcinoma (HNSCC) patients irradiated *ex corpora* need more time to repair DNA double strand breaks (dsbs) than lymphocytes from healthy donors [5] and greater residual DNA damage was detected with the single cell gel electrophoresis (comet) assay in these patients [1,2].

Several studies show that addressing DDR *in vivo* can lead to novel and clinically relevant insights. A non-invasive approach in mouse xenograft tumors revealed a second wave of dsbs, marked by formation of phosphorylated histone variant H2AX (γH2AX) foci, occurring 2 days after the initial wave [6]. The cause of this second, unexpected wave of dsbs is still unknown, with suspected causes of radiation induced genetics instability and apoptosis [6]. DDR studies in *ex vivo* cultures from normal human prostate tissue treated with ionizing radiation (IR) and cytotoxic agents resulted in different responses of basal versus luminal epithelial cells. The latter lacked γH2AX foci formation completely, despite normal 53BP1 foci formation [7]. Recently, some emphasis has been put on *in vivo* DDR studies in patients: (i) Base excision- and nucleotide excision repair (BER/NER) measurements in human colorectal biopsies (neoplastic and adjacent normal tissue), revealed patient- but not tissue-specific repair activity [8]. (ii) A study using normal epithelium of human breast cancer patients concludes that S/G2 cell cycle arrest during the course of radiation therapy (RT) leads to greater use of homologous recombination (HR) [9]. (iii) Analysis of HR defects in sporadic human breast cancer patients showed low RAD51 scores being a strong predictive marker of pathologic complete response to chemotherapy [10]. Taken together, these studies suggest that using DDR activity/proficiency as an *in vivo* readout could lead to a more effective and appropriate treatment of individuals.

Spontaneous tumors in companion animals like dogs have been described to offer a unique opportunity as a model for human cancer biology and translational clinical research [11]. In contrast to many murine tumor xenograft studies, canine tumors develop naturally and grow over long periods of time in the setting of an intact immune system. Human and canine tumors share many similarities, such as inter-patient tumoral heterogeneity, high incidence with age, similar biological behavior concerning development of resistance and metastasis, and comparable responses to antineoplastic agents. Furthermore, several studies indicated that factors of the DDR pathways also affect both disease development and treatment response in dogs [12-14]. As the evolution of most cancers in dogs is shorter than that of humans, conclusions from clinical studies can be drawn faster. Together with the high amount of dog owners willing to participate in clinical studies ([11,15] own experience), the dog could serve as a model to explore the importance of DDR and especially repair kinetics after antineoplastic treatment *in vivo*, thus offering opportunities for both human and animal healthcare. However, so far very little is known about DNA repair mechanisms and DDR in the canine background. Herein, we will critically investigate the current knowledge of canine DDR and discuss its potential to provide a basis as a model for DDR *in vivo*.

## Discussion

Animals spontaneously developing cancer within an intact immune system are proposed to provide an excellent opportunity to investigate various aspects of cancer [16,17]. As opposed to experimental animals, companion animals are genetically outbred and immunologically competent, thus forming cancers that are more similar to human ones in terms of patient size, cell kinetics and heterogeneity. Moreover, clients (owners) are often willing to participate in well-designed clinical trials. Dogs share physiological and metabolic characteristics for most organ systems and drugs with humans and are large enough for multiple sampling opportunities, diagnostic and treatment interventions. Over the last years, several consortia of comparative oncology collaborations have formed and are managed under the National Institutes of Health (NIH)-National Cancer institute’s Comparative Oncology Program (NCI-COP) in order to advance the study of comparative tumor biology and clinical investigations. The yearly cancer mortality rate for dogs < 10 years (deaths due to cancer per 10,000 dog-years-at-risk in Swedish dogs < 10 years) is high with 50% in over 350,000 insured Swedish dogs and varies between breeds [18]. Over 1 million of pet dogs are diagnosed annually and managed with cancer in the United States [16], and these patients can often be entered in clinical trials when conventional treatments do not meet the goals of the veterinary oncologist. Features of certain canine cancers are already well characterized and show similarities with the human situation [17,19]. In the following sections we critically discuss, if - based on the current knowledge - the dog can be proposed as a model to study DDR *in vivo,* as well as point at missing links in this regard.

### Are canine and human genomes similar enough to comparatively study DNA damage response and repair?

The canine genome has been sequenced and is available for studies identifying and associating genetically caused diseases, which are of relevance for both animal and human health. Bioinformatic analyses determined that around 94% of the dog genome belongs to regions of conserved synteny between the dog, human, mouse and rat genomes [20]. The euchromatic part of the canine genome is only about 18% smaller than the human genome [21], but the human and dog genomes differ largely in the chromosome number (46 and 78, respectively). With respect to the common ancestor of eutherian mammals (CAE, 2n = 42), their genome is substantially rearranged. However, mouse and rat genomes are also severely altered with respect to the CAE genome, as they are highly rearranged and have accumulated large numbers of nucleotide substitutions in neutral sites [22]. Nonetheless, the canine gene products seem to be more closely related to their human homologs than those of mice. This suggests potentially higher functional similarity between canine and human proteins, as well as indicates possibly better crossreactivity of human antibodies with canine proteins than with murine ones, especially with respect to DDR proteins ([23]; own observations). The antibody crossreactivity would be especially beneficial in case of functional studies. Humans and dogs share an ancestrally related pathogenic basis for cancer, with pathognomonic genetic changes being conserved in both species [24]. As an example, the *BCR-ABL* fusion gene could be detected with fluorescence in situ hybridization (FISH) in canine chronic myelogenous leukemia (CML) and chronic monocytic leukemia, which is equivalent to the Philadelphia chromosome (with the *BCR-ABL* fusion) in human CML showing equal genomic break sites [24,25]. Besides similarities in protein-coding regions, it is important to keep in mind that the slight differences in the total amount of canine and human genetic material could result in different levels and regulations of the micro (mi) RNAs, which are becoming increasingly relevant. Apart from genetic alterations of proteins, alterations in the miRNA coding regions were shown to affect the regulation of DNA repair [26,27]. Nevertheless, as it is well established that the canine gene products are very similar to the human ones, the *functional* read-outs of canine studies based on the protein-coding regions do exhibit a high potential to result in deeper understanding and more accurate predicting of the treatment-response. Involvement of proteomic screens could provide additional insight in this matter.

### Are alterations in DNA damage response genes relevant for the development of canine cancer?

In transformed tumor initiating cells with continuously activated DDR, throughout mammalian species, deregulated cell cycle check points and apoptosis mechanisms often prevent an efficient halt of proliferation and cell death induction. Amount of evidence clearly demonstrates that the very similar misregulations occur in both humans and dogs, resulting in genomic instability and tumor progression. Abrogation of p53 function by mutational and non-mutational mechanisms is one of the most frequent tumor suppressor gene inactivations in humans and domestic animals, while p53 dysfunction and MDM2 (ubiquitin E3 ligase of p53) overexpression play a central role in cancer progression [28-31]*.* Similarly, p16 an important cell cycle regulator encoded by the gene *CDKN2A* (Cyclin-dependent kinase inhibitors 2A; also called multiple tumor suppressor 1) is often mutated in a variety of human as well as canine cancers [32-34]. Loss of nuclear p16 expression is a prognostic marker for human melanoma and readily described in canine malignant melanoma [32,35,36]. P21, a CDK inhibitor regulating cell cycle progression is frequently down-regulated in both human and canine tumors [37,38]. Consequently, the extent of genomic instability has been described to be equally comparable in certain canine and human tumor types, such as osteosarcoma and colorectal cancer [39,40]. Taken together present finding clearly indicate that alterations in DDR genes are relevant for development of canine cancer, however to shed more light on tumor-associated defects, further investigations of different canine tumor types with regard to their mutational status and in particular the functional effects of mutations are needed.

### Can DNA damage response be compared and transferred between the two species?

Only little is known about the DDR in the normal canine background and its potential alterations in neoplastic tissues. Nevertheless, as discussed bellow, few available studies indicate major similarities between human and canine DDR pathways.

#### DDR initiation

Upon Minute virus of canines (MVC, an autonomous parvovirus) infection classical DDR is triggered in canine cells [23]. ATM activation leads to strong H2AX phosphorylation whereas ATR leads to RPA32 phosphorylation; both of which were also reported to take place in human cells [23]. The MRN complex, which initially recognizes DNA dsbs was additionally visualized. In summary, the ATM-Mre11 axis is induced at the MVC replication centers during infection. To our knowledge the MVC study is the very first example of several important DDR proteins being detected with human antibodies in canine cell lines. Thus suggesting high homology between the proteins of two species. Another study confirmed that the broadly used human antibody against γH2AX is applicable in canine cells as well [41]. Although the MVC study is the only investigation of DDR initiation in canine cells, it: (i) implies similarities between human and canine response and (ii) represents an important starting point for exploring the impact of other stressors on canine cells.

#### DNA dsb repair

The amount of data directly comparing dsb repair kinetics in human and canine cells is very limited. One study addressing the capacity of nuclear extracts to bind a linear DNA probe (mimicking a DNA dsb) [42] revealed that in comparison to human extracts, proteins from canine extracts bind with a much lower affinity to linear DNA (28-fold); proteins from hamster cell extracts exhibited further decreased affinity [42]. The mechanism underlying this discrepancy is however not understood yet. Recent comparison of the dsb repair kinetics by pulse field gel electrophoresis (PFGE) after etoposide treatment indicated that the activity of fast non-homologous end joining (NHEJ) repair is 25% lower in canine than human cells, whereas the slow HR pathway seems to be similar. Unfortunately, in this study the relative ratio of migrated to non-migrated DNA was not taken into account, although it differed significantly between the two species [43]. NHEJ reduction in canine background potentially indicates that DNA-PKcs, main kinase in this pathway, could be more important in the primate background. Indeed the intrinsic activity of this protein is 13-fold lower in canine than in human fibroblasts [44]. The draw back of this study however is that it utilized whole cell extracts, in which the overall amount and activity of DNA-PKcs could be influenced by interspecies differences in the amount of cytosolic proteins or other components. Another potential consequence of increased DNA-PKcs activity in human cells is that DNA-PKcs and its partner Ku could bind faster to the DNA break ends in this background. Consequently, the breaks would be protected faster and more often repaired via classical NHEJ in human cells. As DNA-PKcs also regulates the activity of backup-NHEJ pathways, these might be more active in canine cells with less detectable DNA-PKcs [45]. If NHEJ is less active in canine cells, then HR might be a preferred dsb repair pathway.

**Figure 1:**
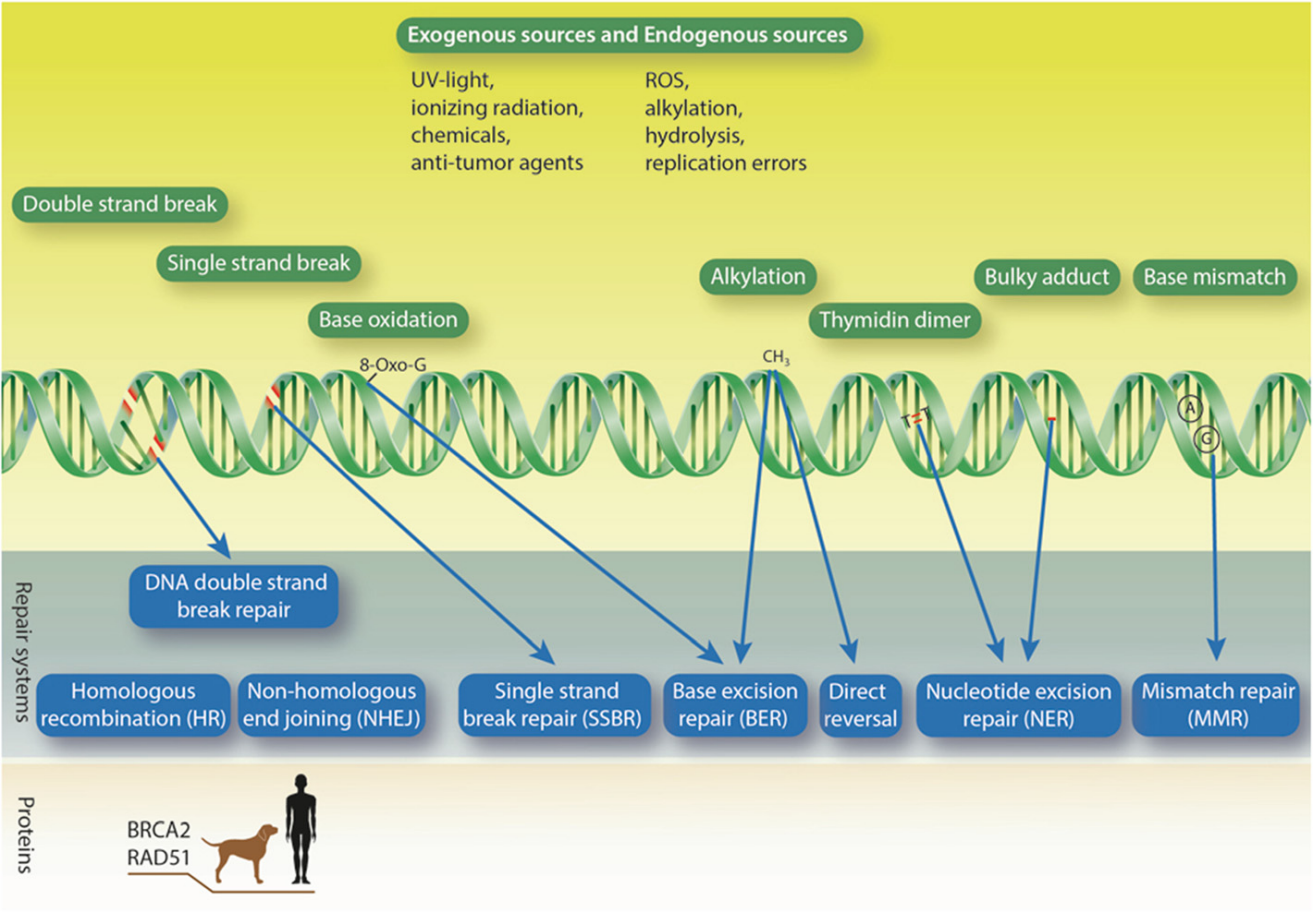
**DNA damages and corresponding repair mechanisms.** Various exogenous and endogenous DNA damaging agents attack the DNA on a daily basis. As a result many different types of DNA lesions are generated (green DNA strand with marked damage types (red or written) and green boxes with names of damage types). In order to survive, the cells harbor a set of repair pathways (blue boxes). Important players mutated or misregulated in both canine and human cancers are depicted in the lower part.

In addition to above described findings of Park et al. suggesting that HR could be equally active in both species [43], mutations in different HR components have been analyzed in tumor setting. As BRCA-mutations lead to a higher risk of developing certain types of cancers in humans, the expression levels of these genes were analyzed in dogs with mammary cancer. In canine mammary carcinomas, BRCA2 and RAD51 show similar regulations, which indicates similar functions (Figure 1). In adenoma vs. normal samples, BRCA2/1 and RAD51 expression was reduced. In more advanced adenocarcinomas, however, BRCA2 and RAD51 were overexpressed in about 50% of the cases. Overexpression was even more pronounced in lymph node metastases [46]. Experimental studies are ongoing to clarify if these changes are a direct response to altered genetic stability or if they spontaneously occur during tumor formation. In English Springer Spaniels with mammary tumors, BRCA1 and BRCA2 genes seem to be involved in the development of the tumor [19]. Furthermore, BRCA1 is possibly involved in the malignant behavior [47]. However, the results are sometimes conflicting and more cases have to be analyzed to draw firm and general conclusions.

Taken together limited amount of data does not allow drawing of strong conclusions about the similarities between dsb repair in humans and dogs. However, there are clear indications that certain pathways such as HR might have higher degree of similarity between the two species. This could be of particular interest in translational research, particularly the one based on synthetic lethality. To fully understand the significance and extent of differences between human and canine NHEJ pathway future studies are needed such as, quantification of phosphorylated DNA-PKcs-foci, NHEJ assays using a pathway-specific substrate, determination of DNA-PKcs protein levels with different antibodies and quantitative mass spectrometry. The activity and efficiency of HR in canine cells needs to be examined in further depth, among others by comparing human and canine Rad51 foci kinetics after the treatment with different genotoxic agents.

#### Base excision repair and nucleotide excision repair

Mechanisms like BER and NER have evolved to preserve the fidelity of the genomic material, which is continuously attacked by endogenous and exogenous stressors (Figure 1). The efficiency of the formerly mentioned repair pathways, especially BER, is thought to correlate with lifespan. Though dogs live shorter than humans (16.6 years vs. 90 years, respectively) [42], the BER capacity of canine and human embryonic fibroblasts under atmospheric oxygen tension (20%) is not significantly different [43]. In contrast to BER, NER activity was shown to be significantly different between the two species (25% lower in dogs) [43]. Performing the assay under physiological oxygen tension (3%), BER activity was also lower in dog cells [43]. These two pathways could therefore vary in activity between the two different species. However, in the case of *in vitro* assays, the salt conditions and redox potentials influenced the reactions massively, which could explain observed repair differences between the species [48]. Furthermore, cellular growth conditions can influence the total BER protein expression [49], rendering direct inter-species comparisons difficult. Interestingly, the activity of DNA polymerase β, the key enzyme in filling a single nucleotide gap during BER, was increased in species with shorter lifespan [50]. Though these findings point at intriguing similarities and differences between human and canine excision pathways, as in case of dsb repair, extensive work is needed to understand to which extent DNA repair is comparable between the two species.

### In which tumors do we have sufficiently based potential to compare DNA damage repair?

#### Breast cancer

Tumor gene expression studies of BRCA-mutations in malignant canine mammary tumors have shown varied results with under-expression of BRCA1 in malignant, as well as over-expression of BRCA2 in metastatic tumors [46,47]. As in women, germline mutations also showed a significantly increased risk of mammary cancer development in the examined breed of English Springer Spaniels [18,19]. BRCA2 and Rad51 expression were proposed as histologic criteria in canine breast cancer staging (Figure 1) [46]. While little is known about the DDR in canine mammary cancer, comparable BRCA2 and Rad51 misregulations, point towards a high possibility of similarly altered HR pathway in the two species.

#### Prostate cancer

Compared to men, the incidence of prostatic cancer in dog is low. However, the spontaneous development of the disease in dogs has awoken the interest to use dogs as a comparative model for prostate cancer [51]. The disease in dog behaves similarly to high-grade prostate cancer in men and – although the highly aggressive variant is rather rare in elderly men - the model character can be exploited for treatment strategies such as chemotherapy, vascular targeting, radiation therapy approaches and management of disseminated disease.

#### Osteosarcoma

Canine osteosarcoma has been shown in many studies to be a valuable comparative model, as it has many similarities on the genetic level, in clinical and biological behavior and in metastasis formation [52,53]. Case collection is more rapid, as osteosarcoma is much more common in dogs than in man. Common genetic and molecular alterations affect p53, retinoblastoma protein (Rb), c-Met, GH and IGF-1 [52]. So far, little is known about DNA repair in canine osteosarcoma. In many DDR studies, the human osteosarcoma cell line U2OS was used and in further studies findings should be compared with canine osteosarcoma cell lines.

#### Skin cancer

Physical factors, such as cumulative exposure to DNA damaging agents, such as UV-radiation, and viral factors, such as papilloma-viruses, have been described as causative agents in canine cutaneous neoplasia. Canine skin tumors may also be induced directly through genetic mutations in factors such as p53 [54,55]. In two of the common malignant tumors of the skin, squamous cell carcinoma and melanoma genes and proteins regulating the cell cycle and cell death are affected. The p53 protein was shown to solely localize to the cytoplasm in many tumor cases [13]. P16 expression was significantly reduced [32]. Both proteins usually cause cell cycle arrest or delay, which provides the time for DNA repair or the induction of apoptosis in the case of heavily damaged cells. Therefore, misregulation of important tumor suppressor genes leads to genomic instability and progression of canine melanoma of the skin [32].

#### Hematologic cancer

Non-Hodgkin’s lymphoma (NHL) represents the fifth leading cause of death due to cancer in humans and the high frequency of malignant lymphoma (7-24% of all canine tumors) in dogs continues to increase as well. Chronic myelogenous leukemia (CML), sporadic Burkitt lymphoma (BL) and chronic lymphocytic leukemia/small lymphocytic lymphoma (CLL) are three well-characterized hematologic cancers that are morphologically similar in both species [24]. The common genetic mutations and altered oncogene or tumor suppressor gene expression, as well as signal transduction alterations (including N-ras, p53, Rb, and p16 cyclin dependent kinase aberrations), have been reported to occur similarly in human lymphomas as well as in dogs [14,56,57]. In human chronic myelogenous leukemia (CML), an aberrant *BCR-ABL* fusion transcript is the hallmark of the disease [24]. The aberration is seen in more than 90% of adult patients [58]. It was demonstrated that expression of BCR-ABL leads to the direct down-regulation of DNA-PKcs [59]. This proteasome-dependent degradation leads to a marked DNA repair deficiency and explains how secondary genetic alterations accumulate in CML. In five cases of canine CML, BCR-ABL translocations could be detected as well, affecting 11 – 34% of the cells [24]. Therefore, tumorigenesis of CML seems to be similar to the human malignancy. In the canine situation however, an additional down-regulation of DNA-PKcs still has to be verified.

In summary the five depicted tumor types are highly adequate models to translationally study tumor biology and treatment responses. We postulate that these tumors can also be used to study the DDR *in vivo*. In many of these tumors, cell cycle control proteins are altered, thus indicating increased genomic instability and DDR defects in spontaneously developing canine tumors.

## Summary

In order to answer the question if studies in dogs have potential and perspective to serve as an *in vivo* model for DDR a positive outlook can be granted. Integrating spontaneous canine tumor models has several important advantages. Due to the high caseloads in veterinary clinics and shorter lifespan, studies can be performed quite fast. Cancers occurring in dogs and humans arise naturally with age, in the background of an intact immune system. They comprise many common features like histological appearance, tumor genetics, molecular targets, biological behavior and response to conventional therapies. Moreover, in many terms a canine model will even serve better than the murine one to study DDR and its defects *in vivo,* as in mice certain repair pathways seem to be less active in comparison to the human mechanisms. Therefore, mice have potentially a different emphasis and hierarchy of DNA repair pathways [43]. As described above, rather little is known about the DDR in canine cells and tissues. However, the antibody cross-reactivities of the human and canine proteins and the findings summarized in this article clearly show that the DDR of dog cells is potentially highly similar to human cells. In order to use canine tumor patients as models, the regulation and kinetics of the canine DDR will have to be studied more thoroughly at the biochemical and cellular level, by gene and mutational analyses as well as by global molecular pathway studies aiming to elucidate the similarities and differences to human cancers. In this way, the dog as our closest companion can help to better understand the DDR *in vivo* and to verify new treatment strategies on the DNA level *in vivo*.

## Abbreviations

ATM: Ataxia telangiectasia mutated; ATR: ATM and ataxia telangiectasia and Rad3-related protein; BER: Base excision repair; BRCA: Breast cancer protein; BCR-ABL: Breakpoint cluster region-Abelson murine leukemia viral oncogene homolog; 53BP1: p53-binding protein 1; CAE: Common ancestor of eutherian; c-Met: MNNG HOS transforming gene; CDKN2A: Cyclin-dependent kinase inhibitors 2A; CIN: Chromosomal instability; CML: Chronic myelogenous leukemia; CLL: Chronic lymphocytic leukemia/small lymphocytic lymphoma; CRC: Colorectal cancer; DDR: DNA damage response; DNA: Deoxyribonucleic acid; DNA-PKcs: DNA-dependent protein kinase, catalytic subunit; dsb: Double strand break; FISH: Fluorescence in situ hybridization; GH: Growth hormone; GHR: Growth hormone receptor; H2AX: Histone variant 2AX; HER2: Human epidermal growth factor receptor 2; HNSCC: Head and neck squamous cell carcinoma; HR: Homologous recombination; IGF-1: Insulin-like growth factor 1; IR: Ionizing radiation; MDM2: Mouse double minute 2 homolog; MnSOD: Manganese superoxide dismutase; MRN: MRE11-RAD50-NBS1; MSI: Microsatellite instability; MVC: Minute virus of canines; Nbs1: Nijmegen breakage syndrome 1; NER: Nucleotide excision repair; NHEJ: Non-homologous end joining; NHL: Non-Hodgkin’s lymphoma; NIH: National institutes of health; PFGE: Pulse field gel-electrophoresis; Rb: Retinoblastoma protein; RPA: Replication protein A; RT: Radiation therapy; SCID: Severe combined immunodeficiency; SSBR: Single strand break repair; UV light: Ultraviolet light.

## Competing interests

The authors declare that they have no competing interests.

## Authors’ contributions

NG: Conception, design and writing. BvL: Conception, design, critical revision. CRB: Conception, design, writing and critical input. All authors read and approved the final manuscript.

## Pre-publication history

The pre-publication history for this paper can be accessed here:

http://www.biomedcentral.com/1471-2407/14/203/prepub
